# Neuronal growth regulator 1 may modulate interleukin-6 signaling in adipocytes

**DOI:** 10.3389/fmolb.2023.1148521

**Published:** 2023-04-28

**Authors:** Ara Yoo, Soojin Lee

**Affiliations:** Department of Microbiology and Molecular Biology, Chungnam National University, Daejeon, Republic of Korea

**Keywords:** neuronal growth regulator 1 (Negr1), interleukin-6 receptor (IL-6R), obesity, adipocytes, depression, signal transducer and activator of transcription 3 (STAT3)

## Abstract

Interleukin-6 (IL-6) is a pleiotropic cytokine that plays both anti- and pro-inflammatory roles. Due to the restricted expression of membrane IL-6 receptor (IL-6R), most pro-inflammatory functions of IL-6 are attributed to its association with soluble IL-6R (sIL-6R). Neuronal growth regulator 1 (NEGR1) is a brain-enriched membrane protein that has recently been recognized as a risk factor for many human diseases including obesity, depression, and autism. In the present study, we report that the expression levels of IL-6 and IL-6R, as well as the phosphorylation of signal transducer and activator of transcription (STAT) 3, were significantly elevated in white adipose tissues of *Negr1* knockout mice. Elevated levels of circulating IL-6 and sIL-6R have also been observed in *Negr1* ^*−/−*^ mice. Furthermore, NEGR1 interacted with IL-6R, which was supported by subcellular fractionation and an *in situ* proximity ligation assay. Importantly, NEGR1 expression attenuated the phosphorylation of STAT3 by sIL-6R, suggesting that NEGR1 negatively regulates IL-6 trans-signaling. Taken together, we propose that NEGR1 may play a regulatory role in IL-6 signaling by interacting with IL-6R, which may contribute to a molecular link underlying obesity, inflammation, and the depression cycle.

## 1 Introduction

Interleukin (IL)-6 is a pleiotropic cytokine that belongs to a four-helix bundle cytokine group that includes IL-27, IL-31, leukemia inhibitory factor (LIF), and neuropoietin (Erta et al., 2012; Rose-John, 2012). In addition, IL-6 contains both pro- and anti-inflammatory elements (Scheller et al., 2011) and has hormone-like attributes that widely influence immune and non-immune cells (Hunter and Jones, 2015). Accordingly, IL-6 has been implicated in diverse human pathologies, including vascular diseases, lipid metabolism, insulin resistance, neuroendocrine diseases, and neuropsychological behavior (Hunter and Jones, 2015).

In target cells, IL-6 binds to the IL-6 receptor (IL-6R/CD126), an 80-kDa type I membrane protein, which then binds to signal transducer glycoprotein 130 (gp130, also known as IL-6R subunit beta or CD130) (Scheller et al., 2011). While gp130 is ubiquitously expressed, IL-6R is present in only a few cells, including hepatocytes, leukocytes, and megalokaryocytes (Rose-John, 2012). Homodimerization of the receptor complex induces the onset of intracellular signaling via Janus kinase (JAK), leading to phosphorylation of signal transducer and activator of transcription (STAT). The process mediated by membrane-bound IL-6R is call classic signaling.

Interestingly, a soluble form of IL-6R (sIL-6R) has been detected in several body fluids with a concentration of 25–35 ng/mL in normal human serum (Wolf et al., 2014). sIL-6R found in human urine and plasma is biologically active by binding IL-6 with an affinity similar to that of membrane-bound IL-6R (Hunter and Jones, 2015). The IL-6/sIL-6R complex can activate a variety of cell types through a second signaling pathway called trans-signaling, owing to the uniformly expressed gp130 transducer receptor (Rose-John, 2012). Importantly, most pro-inflammatory properties of IL-6 are attributed to trans-signaling (Wolf et al., 2014).

Neuronal growth regulator 1 (NEGR1) was originally identified as a differentially expressed gene in human tumor biopsies that is commonly downregulated in many cancer tissues including the colon, ovary, and stomach (Kim et al., 2014). As a glycosylphosphatidylinositol (GPI)-anchored extracellular protein, NEGR1 plays a role in cell-cell recognition and interaction (Kim et al., 2014). NEGR1 is highly expressed in the brain; however, it is also expressed in other peripheral tissue cells including adipocytes, fibroblasts, and endothelial cells (https://www.proteinatlas.org/humanproteome/single+cell+type).

Recently, NEGR1 has been recognized as a risk factor for obesity (Willer et al., 2009) and diverse neurological disorders, including intellectual disabilities, schizophrenia, and major depression (Schizophrenia Working Group of the Psychiatric Genomics, 2014; Sniekers et al., 2017; Dall'Aglio et al., 2021). Our recent studies using *Negr1* ^*−/−*^ mice revealed that NEGR1 is involved in adult neurogenesis by interacting with leukocyte inhibitory factor receptor (LIFR) (Noh et al., 2019). Negr1-deficient mice also show increased fat mass, enlarged adipocytes, a prediabetic phenotype, and skeletal muscle atrophy (Joo et al., 2019). In the present study, we report that NEGR1 interacts with IL-6R and modulates IL-6 trans-signaling in adipocytes. Our findings suggest that NEGR1 may function as a novel regulator of IL-6 signaling in peripheral tissues and the central nervous system.

## 2 Materials and methods

### 2.1 Animals and cell culture

*Negr1* ^*−/−*^ mice (Kim et al., 2017) were maintained at 22°C ± 1 °C and 55% humidity with controlled 12-h light/dark cycles under the guidance of the Chungnam National University Institutional Animal Care and Use Committee. Neuro2A (N2a) cells were kindly provided by Dr. Seongsoo Lee (Gwangju Center, Korea Basic Science Institute, South Korea). N2a, 3T3-L1, HeLa, and 293T cells (Yoo et al., 2022) were maintained in DMEM (Welgene, Gyeongsan, South Korea) supplemented with 10% FBS (Atlas Biologicals, Fort Collins, CO, United States). SKOV-3-FLAG-NEGR1 stable cells (Sim et al., 2022) were cultured in RPMI 1640 medium.

### 2.2 Cloning

The cDNA clone of IL-6R was obtained from Sino Biological Inc. (China) and subcloned into a pcDNA3-3FLAG vector (Joo et al., 2019) using *Not*I and *Xba*I to produce FLAG-tagged IL-6R protein at its N-terminus. The GST-fused IL-6R ectodomain (20-357a. a) construct was generated by subcloning the pEBG vector (Kim et al., 2017) using *BamH*I and *Kpn*I. Recombinant MYC-hyper-IL-6 was prepared by attaching IL-6 (30–212 a. a) and sIL-6R (1–323 a. a) using a flexible linker (Fischer et al., 1997), followed by subcloning into the pcDNA3-MYC vector (Koh et al., 2015). The cDNA of gp130 was obtained by PCR amplification using total RNA from 293T cells and subcloned into pKH3-3HA (Chun et al., 2016) using *Afl*Ⅱ and *Hind*Ⅲ.

### 2.3 Binding assay and immunoblotting

GST pulldown and immunoprecipitation (IP) assays were performed as previously described (Kim et al., 2017). Briefly, cells were lysed in NP-40 cell lysis buffer (50 mM Tris-HCl, pH 7.4, 150 mM NaCl, 1% NP-40, and 5 mM EDTA) and incubated with glutathione-Sepharose 4B beads (GE Healthcare, Piscataway, NJ, United States) or appropriate antibodies for 2 h at 4°C. Immunoblotting using cell lysate or tissue samples was performed as previously described (Cheon and Lee, 2018). The following antibodies were used to visualize specific proteins: FLAG, MYC, and β-actin (Sigma-Aldrich, St. Louis, MO, United States); GFP, IL-6R, gp130, NEGR1, AKT1, and p-AKT1 (Santa Cruz Biotechnology, Santa Cruz, CA, United States); glyceraldehyde 3-phosphate dehydrogenase (GAPDH; Cusabio, College Park, MD, USA); STAT3, p-STAT3 (Cell Signaling Technologies, Beverley, MA, United States), and human Fc (hFc; Gibco, Thermo Fisher Scientific, Waltham, MA, United States).

### 2.4 Isolation of murine bone marrow-derived macrophages and preadipocytes

Bone marrow-derived macrophages were isolated as previously described (Trouplin et al., 2013). Femoral (thigh) and tibial (chin) bones from 6-week-old male mice were isolated and flushed with serum-free MEM (Gibco) using a 26G needle. The collected cells were then treated with RBC (red blood cell) lysis buffer (Sigma-Aldrich) and filtered using a cell strainer. After the cell suspension was centrifuged, the pellet was resuspended in MEM supplemented with M-CSF (Sigma-Aldrich). Primary preadipocytes were isolated from epididymal white adipose tissue (eWAT) of 6-week-old WT and *Negr1* ^*−/−*^ mice as previously described (Joo et al., 2019).

### 2.5 Quantitative reverse transcription (RT)-PCR

Total RNA was isolated from the tissue samples using an RNA extraction kit (Macherey-Nagel, Duren, Germany). Then, quantitative real-time PCR was performed using a Bio-Rad CFX Connect Real-Time PCR Detection System (Bio-Rad, Hercules, CA, USA) with specific primers for IL-1β (forward: 5′-TGA​AGC​AGC TAT GGC​AAC​TG-3′, reverse: 5′-GGG​TCC​GTC​AAC​TTCAA AGA-3′), IL-6 (forward:5′-TCCTCTCTGCAAGAGACTTCC ATCC-3′, reverse:5′-AAGCCTCCGACTTGTGAAGTGGT-3′), IL-6R (forward:5′-CCACGAAGGCTGTGCTGTTT-3′, reverse:5′-GAC AGGGCACCTGGAAGTCA-3′), IL-10 (forward:5′-GATTTTA ATAAGCTCCAAGACCAAGGT-3′, reverse:5′-CTTCTATGCAGT TGATGAAGATGTCAA-3′), GAPDH (forward:5′-ACAACT TTGGCATTGTGGAA-3′, reverse:5′-GATGCAGGGATGATGTT CTG-3′). GAPDH expression was used to normalize the mRNA levels.

### 2.6 Histological analysis and immunofluorescence microscopy

For histological analysis, eWAT sections were fixed in 4% paraformaldehyde and embedded in paraffin blocks. Tissue sections were then incubated with the appropriate primary antibodies for 1 h, followed by incubation with secondary antibodies such as Alexa Fluor 350 anti-rabbit IgG, Alexa Fluor 488 anti-rabbit IgG, Alexa Fluor 568 anti-mouse IgG, or Alexa Fluor 594 anti-mouse IgG antibodies (Invitrogen, Carlsbad, CA, United States). FITC anti-mouse CD80 antibody (Biolegend, San Diego, CA, United States) was used to visualize M1 macrophages. For immunofluorescence microscopy, cells grown on cover-slips were fixed and washed with PBS. After blocking with 10% CAS-block solution (Thermo Fisher Scientific), the cells were incubated with anti-FLAG and anti-IL-6R antibodies. Cholera toxin subunit B Alexa 488 conjugate (Invitrogen) was used as a lipid raft marker. Imaging was performed using an LSM 880 laser scanning confocal microscope with an Airyscan system (Zeiss, Germany) and analyzed using the ImageJ software (National Institutes of Health, Bethesda, MD, United States).

### 2.7 *In situ* proximity ligation assay (PLA)

The PLA assay was performed in SKOV-3-FLAG-NEGR1 cells using Duolink PLA technology reagents (Merck, Darmstadt, Germany), as previously described (Yoo et al., 2022). Briefly, cells were fixed and incubated with anti-FLAG and anti-IL-6R (B-R6; Invitrogen) antibodies for 2 h. After treatment with PLA probes (anti-mouse MINUS and anti-rabbit PLUS) for 1 h, the cells were subjected to a ligation reaction for 30 min. The samples were then incubated with the amplification solution for 2 h and mounted using a mounting medium containing DAPI (Merck).

### 2.8 Raft fractionation, gel filtration chromatography, and binding assay

Lipid raft fractionation was performed using OptiPrep™ iodixanol (Sigma), as previously described (Kim et al., 2014). Briefly, the cell lysate, resuspended in 40% Optiprep, was placed at the bottom of a centrifuge tube and overlaid with a 28% Optiprep solution. After centrifugation at 76,000 ⅹ g for 18 h at 4°C, samples were collected from the top and designated No. 1. Size exclusion chromatography was performed as previously described (Cheon and Lee, 2018). After cells were lysed in size-exclusion buffer (25 mM HEPES, pH 7.4, 150 mM NaCl, 1 mM EDTA, 0.1 mM PMSF, 0.1 mM Na3VO4, 1 mM NaF, and 0.3% CHAPS), the filtered lysates were then loaded on the Sephacryl S-400 HR column (GE Healthcare). Then, the fractions were eluted at a flow rate of 0.8 mL/min.

### 2.9 Measurement of serum IL-6 and sIL-6R level

Blood samples were obtained by retro-orbital bleeding from 12-week-old wild-type (WT) and *Negr1* ^−/−^ mice (*n* = 8). The serum samples were then subjected to ELISA assays for IL-6 and IL-6R using a mouse IL-6 ELISA kit (BD Biosciences, Heidelberg, Germany) and a mouse IL-6R alpha Quantikine ELISA kit (R&D Systems, Minneapolis, MN, United States), respectively.

### 2.10 Statistical analysis

All experiments were conducted independently at least in triplicates, and the results were analyzed using the Microsoft Excel or GraphPad Prism 9 software packages (La Jolla, CA, United States). All values are represented as the mean ± standard error of the mean (SEM). Student’s unpaired *t*-test was employed to determine the significant difference between two data sets, and a *p*-value of <0.05 was considered significant. All statistical analyses are detailed in the figure legends.

## 3 Results

### 3.1 Increased IL-6 mRNA levels in the eWAT of *Negr1* ^−/−^ mice

**FIGURE 1:**
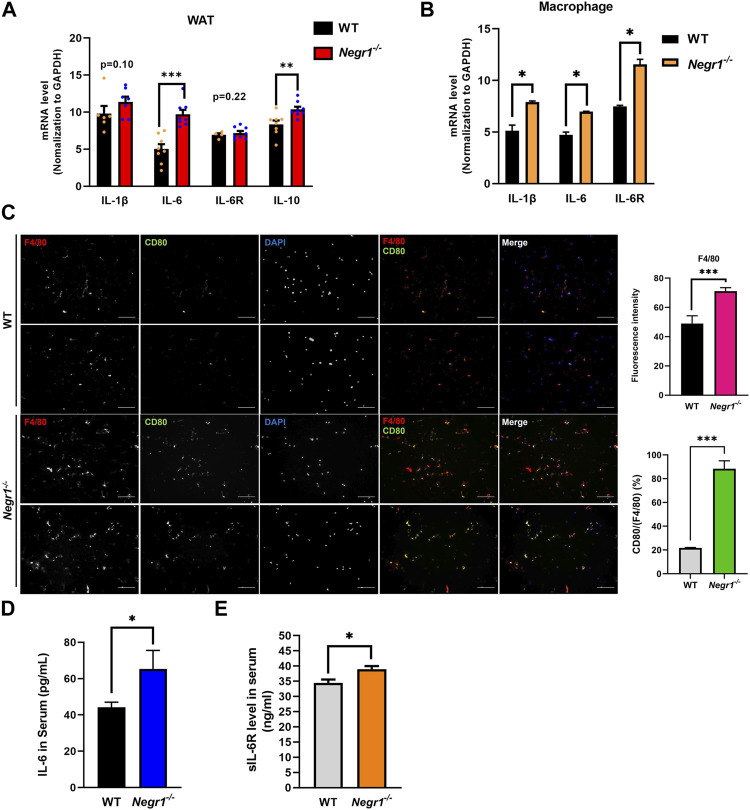
The IL-6 mRNA levels were increased in the eWAT of *Negr1* ^−/−^ mice **(A)** Quantitative RT-PCR was performed to measure the mRNA expression levels of indicated genes using total RNA isolated from the epididymal WAT (eWAT) of 11-week-old WT and *Negr1* ^*−/−*^ C57BL6 mice (*n* = 8). The data represent mean ± SEM. **p < 0.01, ***p < 0.001. GAPDH, glyceraldehyde 3-phosphate dehydrogenase. **(B)** Quantitative RT-PCR analyses using total RNA obtained from bone-marrow-derived macrophages (*n* = 4). *p < 0.05. **(C)** Immunostaining of macrophages in eWAT. The paraffin-embedded WAT tissue sections were incubated with anti-F4/80 (red) and anti-CD80 (green) antibodies. Imaging was performed using an Olympus BX51 microscope. Fluorescence intensities were determined using ImageJ software. ***p < 0.001. **(D, E)** After blood samples were collected from 12-week-old mice (*n* = 8), the levels of IL-6 **(D)** and sIL-6R**(E)** were determined using the ELISA kit. The data represent mean ± SEM. *p < 0.05.

Because obesity contributes to systemic inflammation (Ellulu et al., 2017), and Negr1 knockout mice showed increased WAT adiposity (Joo et al., 2019), we examined the mRNA expression of several cytokines using epididymal WAT (eWAT) of *Negr1* ^−/−^ mice. Among the examined cytokines, IL-6 expression was highly increased in *Negr1* ^−/−^ mice and was approximately 1.9-fold higher than that in WT mice (Figure 1A **).** In contrast, the IL-6R mRNA levels were unaltered. We further investigated IL-6 mRNA expression in primary bone marrow-derived macrophages isolated from *Negr1* ^−/−^ and WT mice. Higher expression levels of both IL-6 (∼1.5-fold) and IL-6R (∼1.7-fold) were observed in macrophages from *Negr1*-deficient mice than those from WT mice (Figure 1B).

Given that macrophage infiltration into adipose tissue is a hallmark of obesity (Bassaganya-Riera et al., 2009), tissue sections of eWAT from WT and *Negr1* ^−/−^ mice were prepared and stained with anti-F4/80 (pan-macrophage marker) and anti-CD80 (M1 macrophage marker) antibodies. An increased number of macrophages (∼1.4-fold) was detected in the eWAT of *Negr1* ^−/−^ mice, indicating increased macrophage infiltration (Figure 1C). In particular, the percentage of CD80-postive cells among the total macrophages were significantly increased in *Negr1*-deficient mice, suggesting that the infiltrated macrophages were more likely to be M1 macrophages.

Finally, plasma IL-6 levels were measured in the blood samples using a mouse IL-6 ELISA kit. IL-6 levels in circulation were ∼1.5-fold higher in *Negr1*-deficient mice than in WT mice (Figure 1D). Moreover, we also examined circulating sIL-6R levels using an IL-6R ELISA kit and found that plasma sIL-6R levels were slightly increased in *Negr1* knockout mice by approximately 1.2-fold compared to WT mice (Figure 1E), suggesting that IL-6 trans-signaling might be enhanced in *Negr1* ^−/−^ mice.

### 3.2 Activation of IL-6/STAT3 signaling was observed in the eWAT of *Negr1* ^−/−^ mice

**FIGURE 2:**
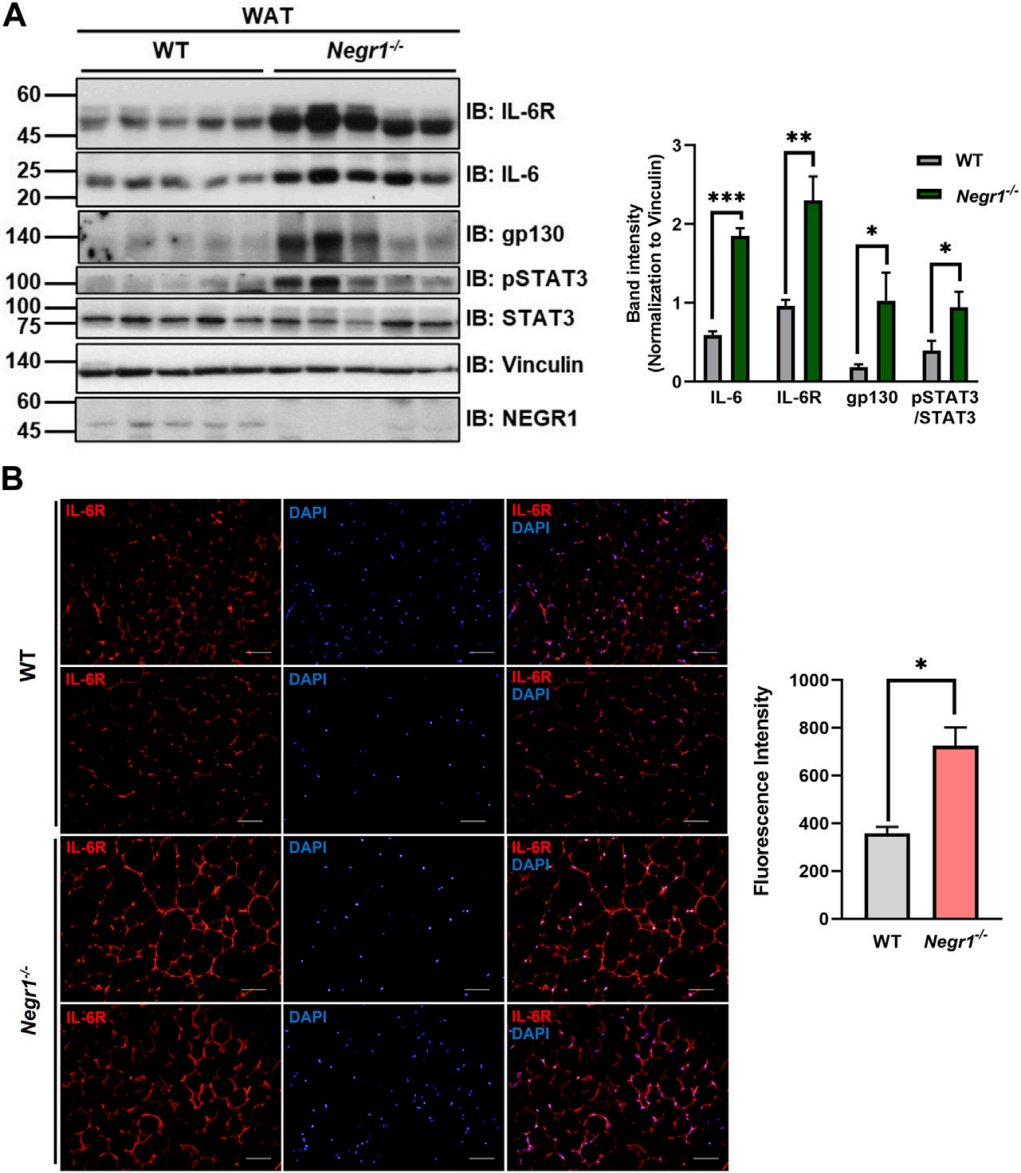
The protein expression levels of IL-6R components in the eWAT of *Negr1* ^−/−^ mice. **(A)** Comparison of protein levels of IL-6 signaling complex in the gonadal WAT of WT and *Negr1* ^*−/−*^ mice (*n* = 9). The band intensity of each protein was normalized to the vinculin level. The data represent mean ± SEM. *p < 0.05, **p < 0.01, ***p < 0.001. **(B)** Visualization of IL-6R in the eWAT of 13-week-old WT and *Negr1* ^−/−^ mice by immunostaining paraffin-embedded tissue sections using an anti-IL-6R antibody (×400 magnification). Fluorescence signals were measured using ImageJ software.

Since the mRNA expression level of IL-6 increased in the eWAT of *Negr1* ^−/−^ mice, we examined the protein levels of IL-6 signaling components in these tissues. Immunoblotting using tissue lysates revealed that the protein levels of both IL-6 and IL-6R were increased by ∼ 3.1- and ∼2.4-fold, respectively, compared to WT (Figure 2A). To confirm the increase in IL-6R levels in *Negr1* ^−/−^ mice, we immunostained eWAT tissue sections from WT and *Negr1* ^−/−^ mice using an anti-IL6R antibody. Combined fluorescence intensities revealed a ∼1.8-fold increase in *Negr1* ^−/−^ mice compared to WT mice (Figure 2B).

Furthermore, the protein level of gp130, the signal-transducing co-receptor, was also highly increased in *Negr1* ^−/−^ mice (∼4.9-fold), indicating that the protein levels of IL-6R complex components were elevated in the WAT of *Negr1*-deficient mice. In addition, pSTAT3 levels were increased in these tissues. Interestingly, samples with higher pSTAT3 levels also showed higher expression of IL-6R and gp130.

**FIGURE 3:**
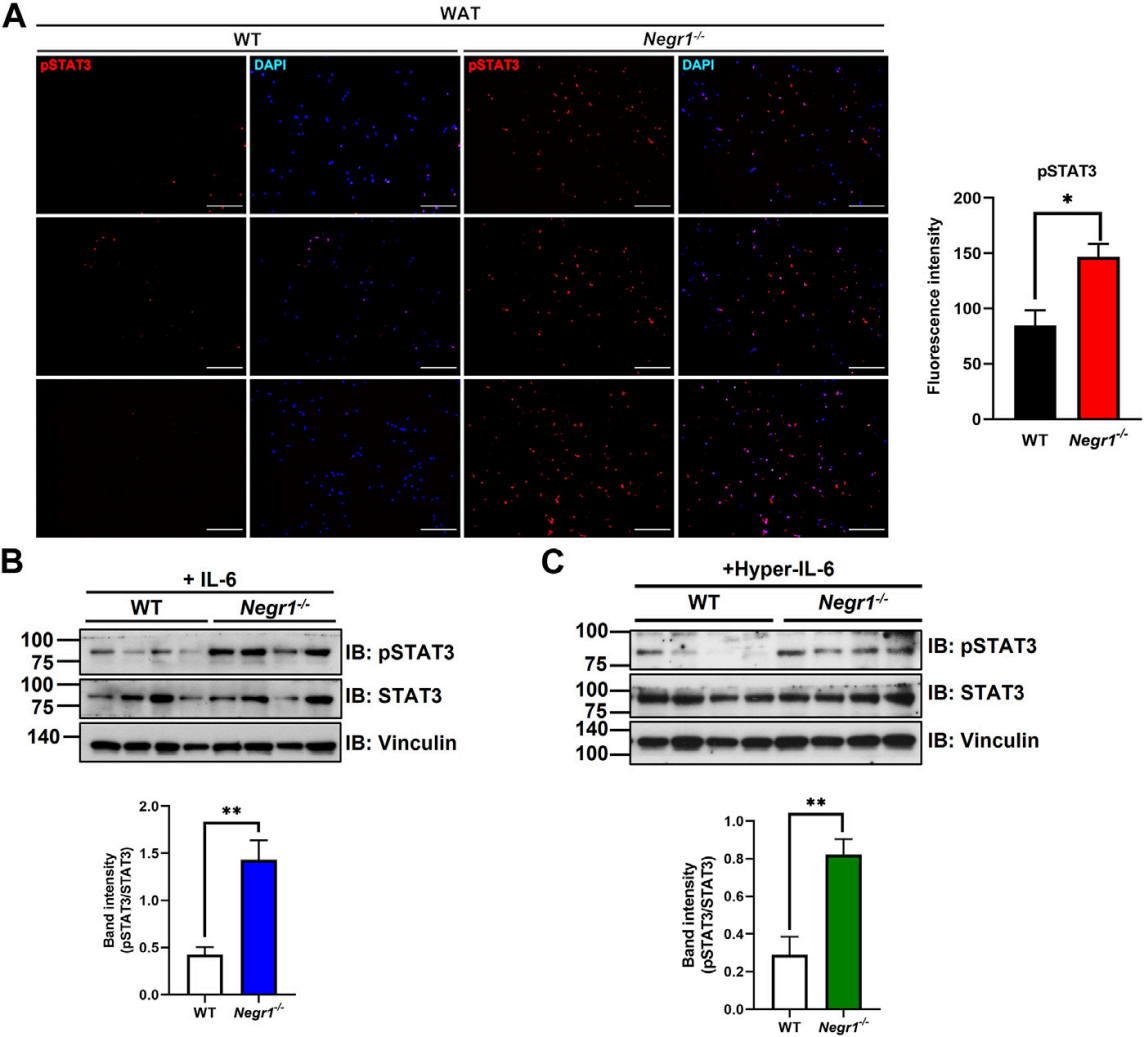
Increased STAT3 activation in the eWAT of *Negr1* ^−/−^ mice. **(A)** Immunostaining of eWAT tissue sections with an anti-pSTAT3 antibody. Imaging was performed using an Olympus BX51 microscope. Fluorescence intensity was calculated using ImageJ software. *p < 0.05. **(B)** Primary adipocytes (*n* = 4) were isolated from WT and *Negr1* ^−/−^ mice and incubated with IL-6 (25 ng/mL) for 10 min before immunoblotting. **(C)** Primary adipocytes were incubated with hyper-IL-6-containing conditioned media for 10 min and pSTAT3/STAT3 levels were calculated based on the band intensities using ImageJ software. **p < 0.01.

The highly conserved STAT signaling pathway plays an important role in the regulation of gene expression in adipocytes (Zatterale et al., 2019). To validate the increased pSTAT3 levels in the WAT of *Negr1* knockout mice, immunofluorescence staining was performed using eWAT tissue sections with an anti-pSTAT3 antibody. The fluorescence signals were approximately 1.7-fold higher in the adipose tissues of *Negr1* ^−/−^ mice than in those of WT mice (Figure 3A).

To further examine the activation of STAT3 signaling in the Negr1-deficient cells, primary adipose cells were isolated from the epididymal fat pads of WT and *Negr1* ^−/−^ mice. In addition, we generated a designer cytokine hyper-IL-6, a fusion protein of IL-6 and sIL-6R connected by a flexible linker (Fischer et al., 1997). After primary adipose cells were incubated with IL-6 (Figure 3B) and hyper-IL-6 (Figure 3C), pSTAT3/STAT3 was calculated based on immunoblotting. In both cases, the pSTAT3/STAT3 level of Negr1-deficient cells was approximately 3.4-fold higher than that of the WT control. Taken together, adipose cells in Negr1-deficient mice exhibited highly enhanced IL-6/STAT3 signaling.

### 3.3 NEGR1 may interact with IL-6R

**FIGURE 4:**
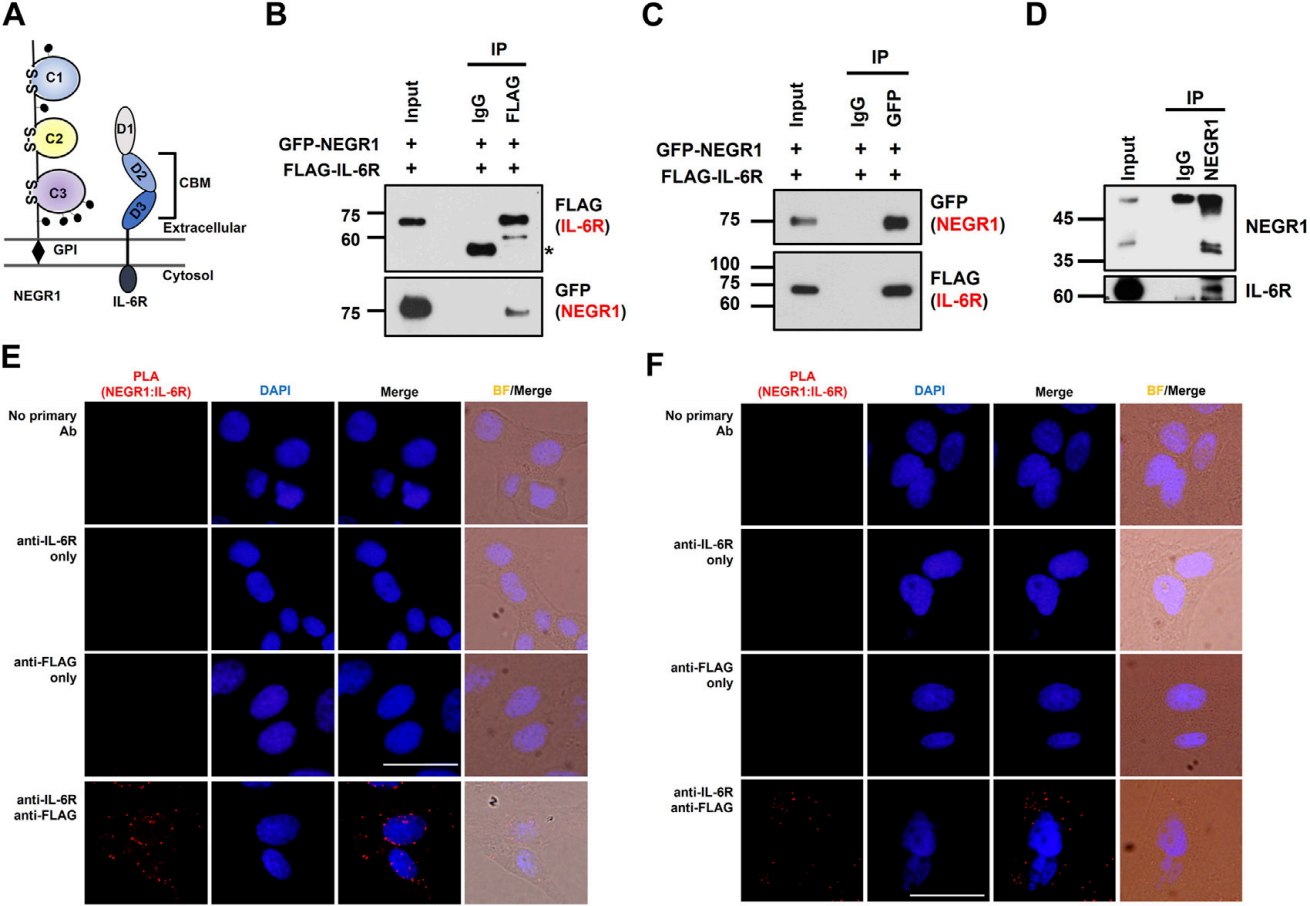
NEGR1 may interact with IL-6R. **(A)** Protein structure of NEGR1 and IL-6R. **(B)** Co-immunoprecipitation (IP) was performed using an anti-FLAG antibody after HeLa cells were co-transfected with GFP-NEGR1 and FLAG-IL-6R for 24h. Co-isolated NEGR1 was visualized with the anti-GFP antibody. **(C)** Reciprocal co-IP between GFP-NEGR1 and FLAG-IL-6R using anti-GFP antibody. **(D)** Co-IP between the endogenous proteins. After HeLa cell lysates were subjected to IP with an anti-NEGR1 antibody, IL-6R was visualized with IL-6R antibody. **(E, F)** *In situ* proximity ligation assay of SKOV-3-FLAG-NEGR1 stable cells under permeabilized **(E)** or non-permeabilized **(F)** conditions. For cell permeabilization, cells were treated with 0.1% Triton X-100 for 10 min. Then, cells were incubated with rabbit anti-FLAG antibody and mouse anti-IL-6R antibody for 1 h, followed by treatment of PLA probes (anti-mouse MINUS and anti-rabbit PLUS). The bar represents 50 μm.

Considering that NEGR1 is a membrane protein facing the extracellular space (Figure 4A), we first examined whether NEGR1 may bind with IL-6 using co-immunoprecipitation (IP), but could not observe binding between these two proteins (data not shown). Next, to determine whether NEGR1 associates with IL-6R, we generated a FLAG-IL-6R construct and transfected it into 293T cells together with pEGFP-C1-NEGR1 (Kim et al., 2014). Subsequently, co-immunoprecipitation was performed using an anti-FLAG antibody, revealing that GFP-NEGR1 was co-isolated with FLAG-IL-6R (Figure 4B). Reciprocally, FLAG-IL-6R co-fractionated with GFP-NEGR1 (Figure 4C).

To examine the interaction between endogenous proteins, we carried out IP with an anti-NEGR1 antibody using HeLa cell lysates. We used HeLa cells because the endogenous NEGR1 protein is readily detected in these cells, whereas it is hardly observed in other cell lines, including 3T3-L1. Although the enriched NEGR1 proteins partly overlapped with the IgG heavy chain bands, we detected co-isolated IL-6R protein in the NEGR1-enriched fraction (Figure 4D), supporting their interaction at the endogenous level.

To demonstrate the interaction between NEGR1 and IL-6R in cells, a proximity ligation assay (PLA) was performed using SKOV-3-FLAG-NEGR1 stable cells (Yoo et al., 2022). In either permeabilized (Figure 4E) or non-permeabilized conditions (Figure 4F), no signals were detected with anti-FLAG (PLUS) or anti-IL-6R (MINUS) antibodies only (2^nd^ and 3^rd^ rows of Figures 4E, F). However, clear fluorescent signals were identified when cells were treated with both antibodies (4^th^ rows), indicating that NEGR1 interacts with IL-6R inside the cell (Figure 4E) as well as at the cell surface (Figure 4F).

### 3.4 Determination of important regions for IL-6R-NEGR1 interaction

**FIGURE 5:**
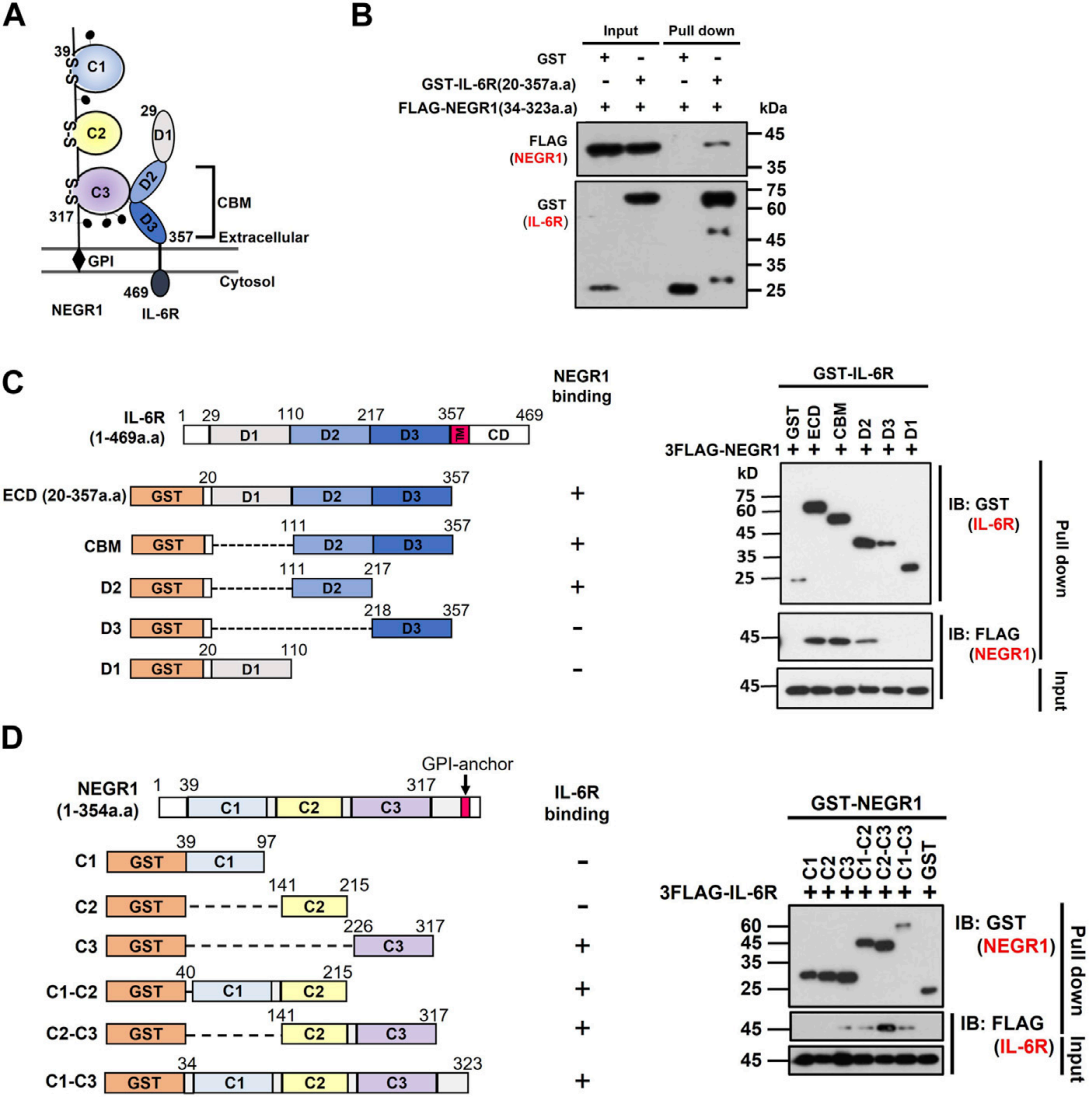
Domain mapping of the interaction between NEGR1 and IL-6R. **(A)** Domain structure of NEGR1 and IL-6R. **(B)** Interaction between GST-fused IL-6R extracellular domain (ECD, 20–357) and FLAG-tagged soluble NEGR1 (34–323) was examined by GST-pulldown. **(C)** Various IL-6R deletion constructs were generated and GST-pulldown was performed after 293T cells were transfected with IL-6R deletion mutants together with FLAG-NEGR1 (34–323). CBM, cytokine binding module; D1 ∼ D3, domain 1–3; TM, transmembrane domain; CD, cytoplasmic domain. **(D)** Determination of NEGR1 domain required for IL-6R interaction. GST-pulldown was carried out after 293T cells were co-transfected with NEGR1 domain mutants and FLAG-IL-6R (20–357). C1 ∼ C3, C2-type immunoglobulin domain 1–3.

While the IL-6R is a typical type-I transmembrane protein, NEGR1 is localized extracellularly (Figure 5A). To determine the critical region for NEGR1-IL-6R interaction, we performed domain mapping experiments using the extracellular region of IL-6R. We first evaluated the interaction between the GST-fused extracellular domain (ECD) of IL-6R (amino acids 20–357) and FLAG-tagged NEGR1 (amino acids 34–323) lacking the GPI-anchoring region (Kim et al., 2014). GST-pulldown showed that NEGR1 was co-fractionated with GST-IL-6R (ECD), but not with the GST control (Figure 5B).

Considering that the IL-6R ECD domain consists of an immunoglobulin (Ig)-like domain (D1) and cytokine-binding module (CBM) domains (D2 and D3) from the N-terminus (Varghese et al., 2002), a series of deletion constructs (CBM, D1, D2, and D3) of GST-IL-6R were generated (Figure 5C). 293T cells were then co-transfected with FLAG-NEGR1 (34–323) and various IL-6R deletion mutant constructs. Subsequent GST-pulldown revealed that NEGR1 was co-fractionated with CBM (D2 and D3) and D2 deletion constructs, indicating that IL-6R D2 was sufficient to interact with NEGR1.

Reciprocally, to determine the NEGR1 binding region, we constructed a plasmid expressing FLAG-IL-6R ECD (20–357). It was then transfected into 293T cells along with various GST-fused NEGR1 deletion constructs (Kim et al., 2017). Among the three consecutive C2-type Ig-like domains (C1, C2, and C3 from the N-terminus), the C2-C3 construct showed strong interaction with IL-6R (Figure 5D). As for the single-domain mutants, only C3 showed binding affinity, suggesting that the NEGR1 C3 region may play a key role in the interaction with IL-6R.

### 3.5 Co-localization analysis of IL-6R and NEGR1

**FIGURE 6:**
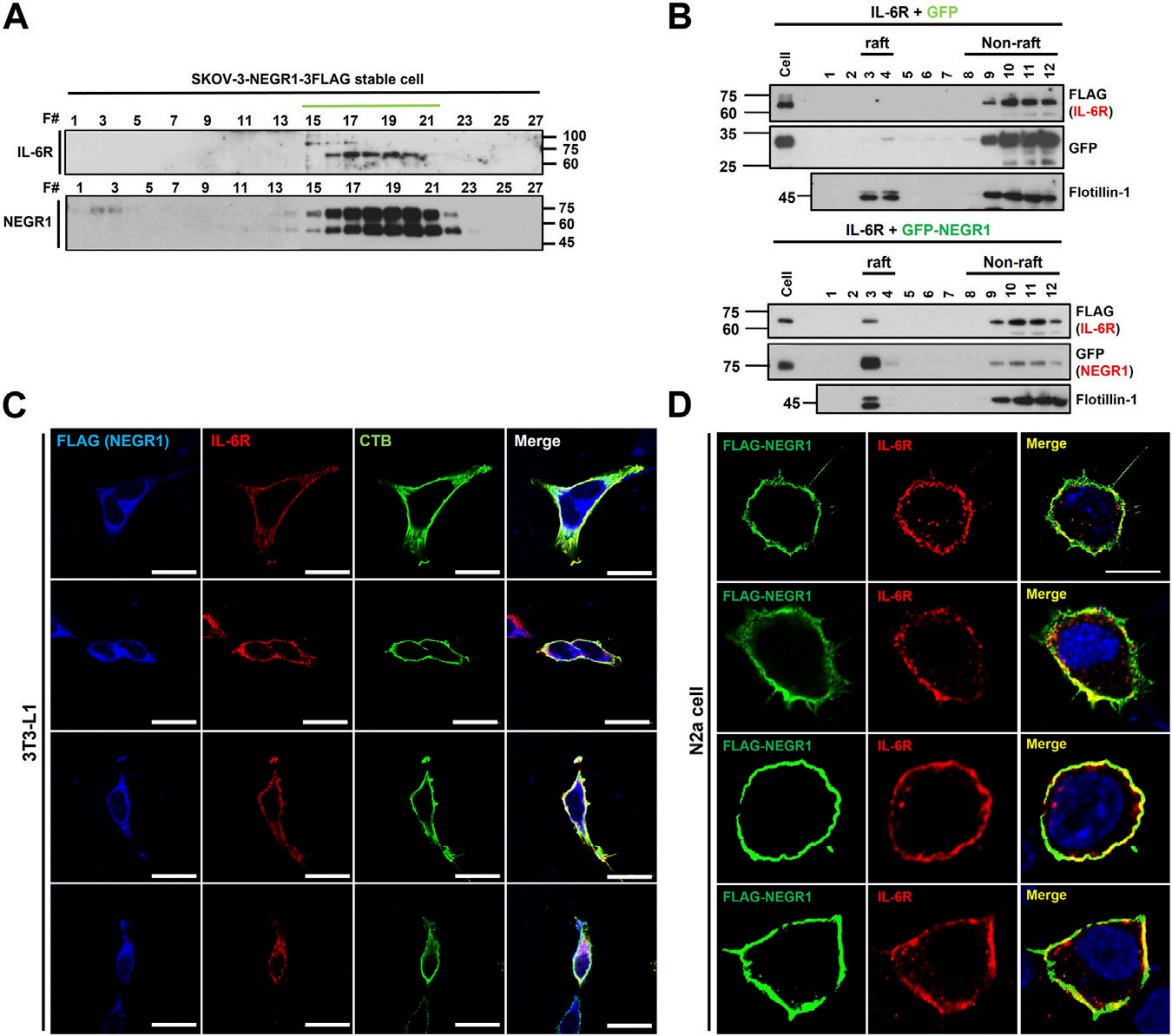
Co-localization analysis of NEGR1 and IL-6R. **(A)** Gel filtration chromatography was performed with the Sephacryl S-400 HR column using SKOV-3-NEGR1-FLAG cells. Eluents were used for immunoblotting with anti-FLAG and anti-IL-6R antibodies. **(B)** Lipid raft fractionation using OptiPrep™ Gradient. Centrifugation was performed at 76,000 × g for 18 h at 4°C after 293T cells were transfected with FLAG-IL-6R together with GFP-NEGR1 (right) or GFP control (left). Frotillin-1 was used as a lipid raft marker. **(C)** Co-localization of NEGR1 and IL-6R in 3T3-L1-FLAG-NEGR1 stable cells. After cell permeabilization, cells were incubated with anti-FLAG (blue) and anti-IL-6R (red) antibodies. Cholera toxin subunit B (CTB) was used to visualize lipid rafts (green). Imaging was performed using a confocal laser scanning microscope Zeiss LSM 880 (Zeiss, Germany). The scale bar represents 50 μm. **(D)** Confocal microscopy of N2a cells after transfection of FLAG-NEGR1 plasmids. Cells were incubated using anti-FLAG (green) and anti-IL-6R (red) antibodies, and cell nuclei were stained with DAPI. The scale bar represents 50 μm.

To evaluate the intracellular distribution of IL-6R and NEGR1, we performed gel filtration chromatography on SKOV-3-FLAG-NEGR1 stable cells using a Sephacryl S-400 HR column. Both proteins were visualized by immunoblotting with anti-IL-6R and anti-FLAG antibodies. The peaks of IL-6R and NEGR1 overlapped closely in gel filtration chromatography (Figure 6A), suggesting that these proteins were present in the same protein complex.

Since NEGR1 is highly enriched in membrane rafts (Kim et al., 2014), we examined whether IL-6R is co-localized in this membrane subdomain. Lipid raft fractionation was carried out after 293T cells were transfected with FLAG-IL-6R plasmid together with GFP-NEGR1 or GFP control. As expected, most of the GFP-NEGR1 protein was present in the raft fraction (Figure 6B, right), whereas frotillin-1 was used as a raft marker. Although no IL-6R was observed in the raft fraction (Figure 6B, left), a small amount of IL-6R protein was found in this subdomain when NEGR1 was co-expressed (Figure 6B, right). This result suggests that some IL-6R proteins were recruited to the raft fraction by NEGR1.

Next, we examined the cellular localization of the two proteins in 3T3L1-FLAG-NEGR1 stable cells. After the cells were immunostained under permeabilized conditions with anti-FLAG and anti-IL-6R antibodies, images were obtained using confocal microscopy (Figure 6C). In addition, cholera toxin subunit B (CTB) was used to visualize lipid rafts. Overlapped signals were detected on both the cell surface and the intracellular region in these preadipocyte cells, which were overlapped well with the CTB. We also visualized the NEGR1 protein in N2a neuroblastoma cells after transfection with the FLAG-NEGR1 plasmid (Figure 6D). When double-immunostained with anti-FLAG and anti-IL-6R antibodies, both proteins were observed prominently at the boundary of the cells, demonstrating their co-localization in the plasma membrane of N2a cells.

### 3.6 NEGR1 may inhibit IL-6 trans-signaling

**FIGURE 7:**
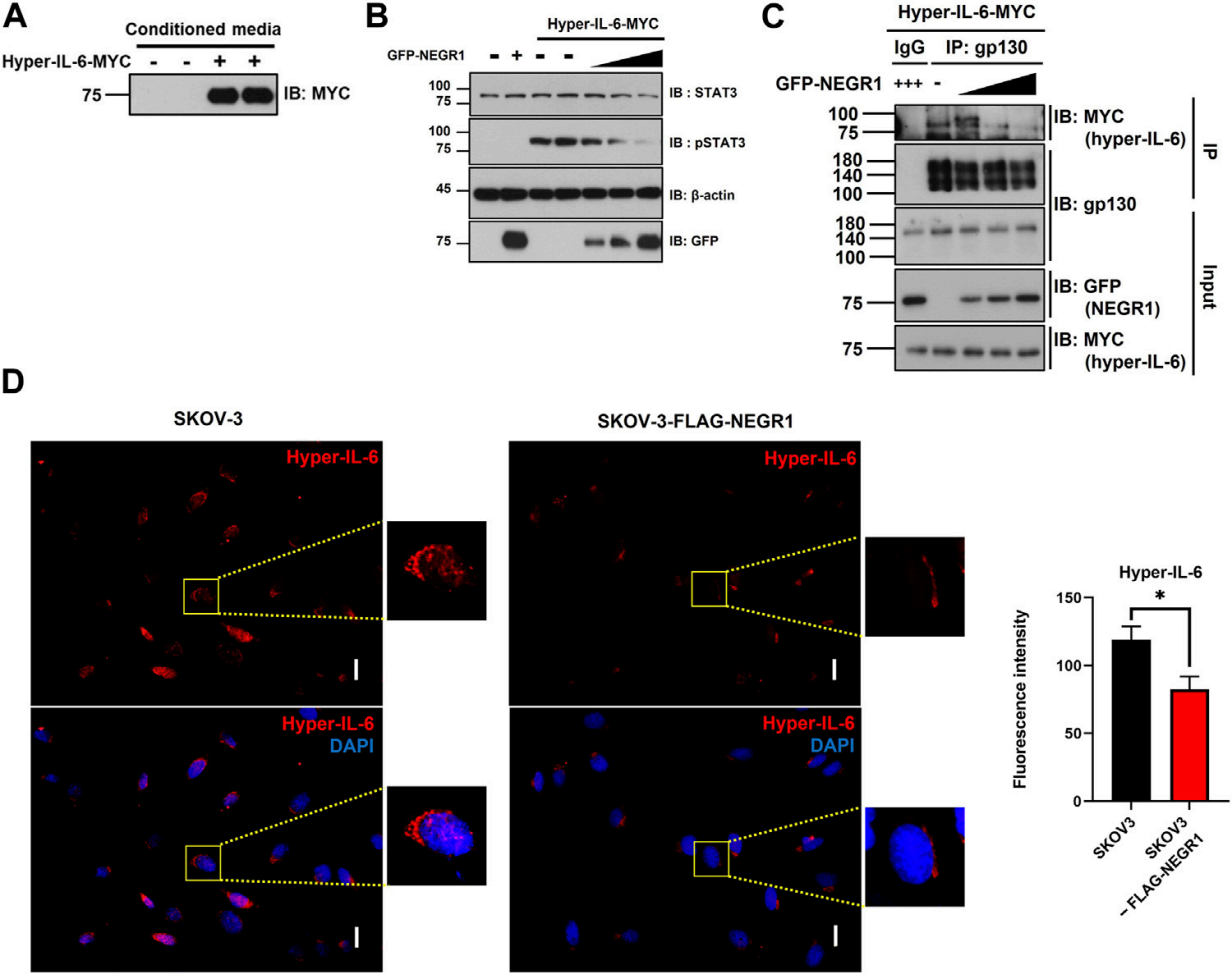
NEGR1 may attenuate IL-6 trans-signaling. **(A)** Hyper-IL-6-containing conditioned medium was obtained after transfection of HeLa cells with pcDNA3-hyper-IL-6-MYC for 24h. **(B)** After transfection with GFP-NEGR1, HeLa cells were incubated with Hyper-IL-6-containing medium for 10 min. Then, cells were lysed and used for immunoblotting with indicated antibodies. Band density was calculated using ImageJ software. **(C)** After HeLa cells were transfected with increasing amounts of GFP-NEGR1 plasmids, the cell lysates were mixed with the hyper-IL-6-containing conditioned medium. Then, IP was performed using an anti-gp130 antibody, and the co-isolated hyper-IL-6 was detected with an anti-MYC antibody. **(D)** Comparison of binding of Hyper-IL-6-MYC on the cell surface. After SKOV-3 (upper panels) and SKOV-3-FLAG-NEGR1 stable cells (lower panels) were incubated with hyper-IL-6 containing conditional medium 1h, cell surface-bound hyper-IL-6 protein was visualized using anti-MYC. Imaging was performed using an Olympus BX51 microscope (Tokyo, Japan). Integrated fluorescence intensity was quantified using ImageJ software. *p < 0.05. Scale bar = 50 mm.

Next, we attempted to determine whether NEGR1 affected intracellular IL-6 signaling. Because we repeatedly noticed that cellular IL-6R levels were influenced by NEGR1 co-expression, we examined IL-6R protein levels after co-transfection of NEGR1 in 293T cells. The IL-6R expression level gradually decreased in accordance with the co-expressed NEGR1 levels (Supplementary Figure S1). Considering that the cellular IL-6R levels are changed by NEGR1 expression and that IL-6 trans-signaling is regarded as a more potent activator of intracellular signaling than classic signaling (Reeh et al., 2019), we focused on IL-6 trans-signaling. To mimic IL-6 trans-signaling, we used a hyper-IL-6 construct, a connected form of sIL-6R and IL-6 fused with a C-terminal MYC tag. HeLa cells were transfected with the hyper-IL-6 construct for 24 h and the culture medium was collected. Secreted hyper-IL-6 protein was confirmed by immunoblotting with an anti-MYC antibody (Figure 7A).

To determine whether the overexpression of NEGR1 influences the IL-6 trans-signaling, HeLa cells were transfected with GFP-NEGR1 along with vector control, and then incubated with hyper-IL-6-containing conditioned medium for 10 min at 37 °C. While the levels of both STAT3 and pSTAT3 were not changed by NEGR1 transfection (2^nd^ lane, Figure 7B), the pSTAT3 level dramatically increased after treatment with hyper-IL-6 (3^rd^ and 4^th^ lanes). When NEGR1 was co-expressed, the hyper-IL-6-mediated pSTAT3 activation gradually decreased in proportion to the NEGR1 expression (5^th^ ∼7^th^ lanes, Figure 7B), suggesting that NEGR1 may suppress IL-6 trans-signaling.

Given that signal-transducing co-receptor gp130 functions to form an active IL-6 receptor complex, thereby initiating intracellular signaling, we examined whether NEGR1 affects the association between gp130 and hyper-IL-6R. After transfection with GFP-NEGR1, HeLa cells were incubated in the hyper-IL-6-containing medium for 10 min at 4°C. IP was conducted using an anti-gp130 antibody. While hyper-IL-6 was not found in the control IgG-enriched fraction, it was co-fractionated with gp130 (1^st^ and 2^nd^ lanes, Figure 7C), demonstrating a specific interaction between hyper IL-6 and gp130. However, gp130-bound hyper-IL-6 levels were reduced in proportion to NEGR1 co-expression (3^rd^ ∼ 5^th^ lanes, Figure 7C).

**FIGURE 8:**
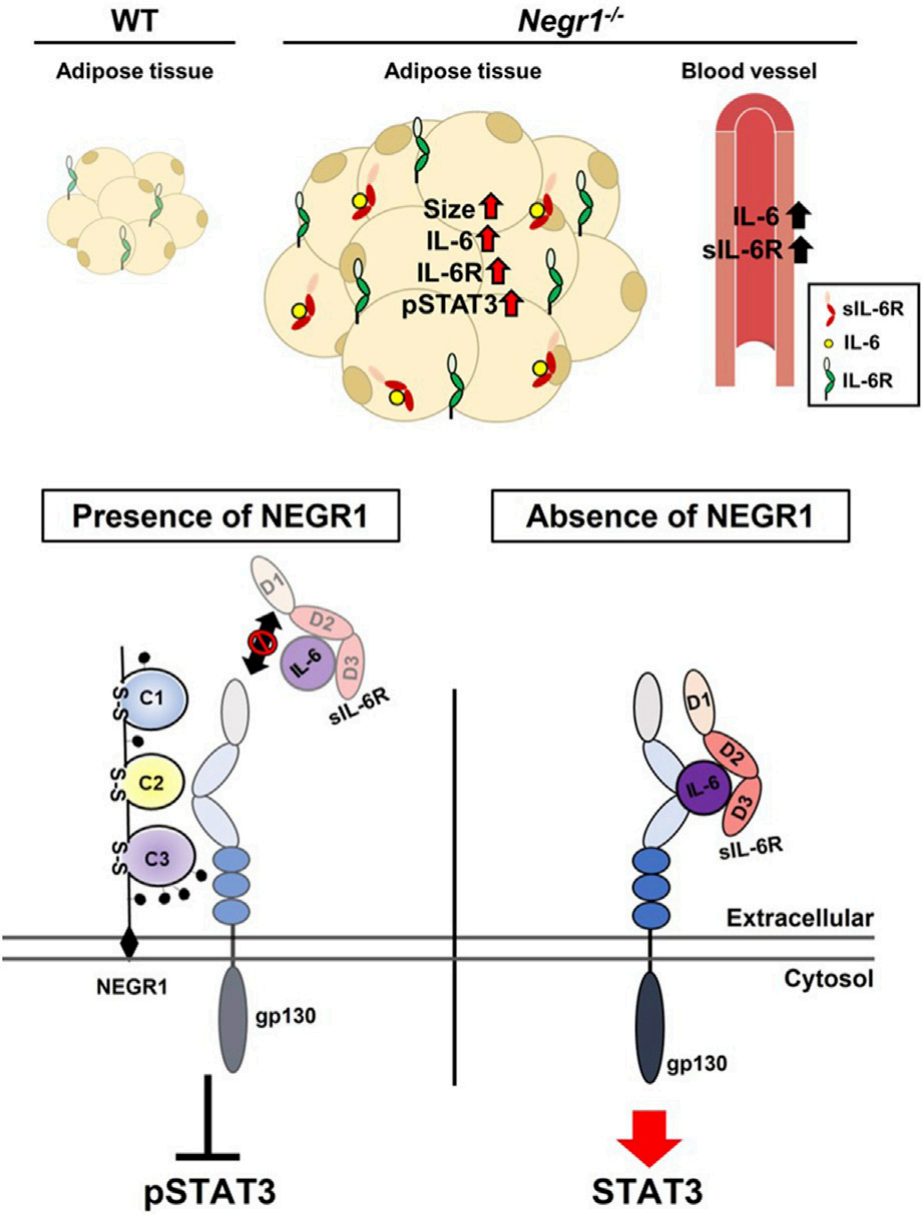
Proposed role of NEGR1 in IL-6 signaling.

To validate these findings, SKOV-3-FLAG-NEGR1 stable cells were incubated in the hyper-IL-6-containing conditioned medium for 10 min at 4°C. Cells were thoroughly washed with PBS, and immunostained with an anti-MYC antibody to visualize cell-bound hyper-IL-6. The combined fluorescence intensity of hyper-IL-6 in SKOV-3-FLAG-NEGR1 cells was approximately 70% of that of the control (Figure 7D). Altogether, we propose that membrane NEGR1 attenuates IL-6 trans-signaling by interfering with the formation of a functional IL-6 receptor complex in the cell membrane (Figure 8).

## 4 Discussion

Among the two major types of adipose tissue, WAT accounts for the vast majority of adipose tissue in humans and is the main site of energy storage (Eder et al., 2009). WAT is also an important source of circulating IL-6, considering that IL-6 levels decrease by approximately 40% in adipocyte-specific IL-6 knockout mice (Han et al., 2020). Although IL-6 is regarded as a systemic regulator of body weight, lipid metabolism, and glucose uptake (Sindhu et al., 2015), the role of IL-6 in obesity remains controversial. The expression of IL-6 and IL-6R is elevated in obese individuals and correlates positively with body mass index and percent body fat (Sindhu et al., 2015). In contrast, adipocytes show increased lipolysis when incubated with IL-6 (Trujillo et al., 2004). Moreover, IL-6-deficient mice showed variable obesity phenotypes between studies, which might be partially correlated with the complex etiology of obesity (Eder et al., 2009).

Previously, we reported that the highly increased WAT with enlarged cell size (hypertrophy) was the most phenomenal characteristic during the anatomical analysis of *Negr1* knockout mice (Joo et al., 2019). Given that obesity is considered a chronic state of low-grade inflammation (Zatterale et al., 2019), it was not surprising to observe considerable increases in the expression of inflammatory cytokines, IL-1 and IL-6, in adipocytes and macrophages in *Negr1* ^*−/−*^ mice (Figures 1A, B). The increased number of macrophages in the WAT of *Negr1* ^*−/−*^ mice (Figure 1C) is also consistent with a typical change during obesity in which monocytes progressively infiltrate adipose tissue (Wellen and Hotamisligil, 2003). However, IL-6R expression levels were not expected to increase in either WAT (Figures 2A, B) or macrophages (Figure 1B), as well as in the serum of *Negr1* knockout mice (Figure 1E). It is well acknowledged that in contrast to soluble receptors of cytokines, such as IL-1α or TNFα, which act as antagonists, no antagonistic effect of sIL-6R has been described, rather mediating the signal transduction of their ligands (Lokau et al., 2016). Therefore, increased serum sIL-6R levels are likely to affect the entire body of *Negr1* ^*−/−*^ mice to enhance IL-6 trans-signaling.

IL-6 serum concentration, which is usually in the picogram per milliliter range in humans, can rapidly increase up to 1000-fold under certain pathological conditions, including inflammatory diseases and cancer (Baran et al., 2018). In contrast, plasma sIL-6R levels are much higher than those of IL-6 and only moderately change during inflammation (Baran et al., 2018). Moreover, the regulatory mechanisms of IL-6R expression are largely unexplored (Lokau et al., 2016), except that the phosphatidylinositol-3-kinase (PI3K)/AKT and mammalian target of rapamycin (mTOR) signaling pathways may regulate IL-6R expression (Garbers et al., 2013). However, the promoter region of gp130 contains STAT1/3 binding sites; subsequently, gp130 expression can be induced by several cytokines including IL-6 (Schmidt-Arras and Rose-John, 2021). The elevated gp130 protein levels in the WAT of *Negr1* ^*−/−*^ mice (Figure 2A) may be associated with altered IL-6 expression and activation of STAT3 signaling in these cells.

The extracellular region of IL-6R is composed of N-terminal Ig (D1), followed by a classical CBM in which domains D2 and D3 are connected at approximately 90° (Varghese et al., 2002). The formation of the signaling-competent IL-6 receptor complex is believed to occur sequentially (Boulanger et al., 2003). IL-6 is first engaged by IL-6R and then associated with gp130 to form an interlocking hexameric structure with three distinctive binding sites (sites I, II, and III). Although IL-6 binds to the elbow region at the junction of D2 and D3 of IL-6R (site I), the majority of the contact surfaces are located in the D3 domain (Boulanger et al., 2003). In addition, the IL-6R D3 domain participates in the interaction with gp130 to form a site II epitope.

However, X-ray analysis has revealed that the tip of the gp130 N-terminal domain forms a large interaction surface with the D2 domain of IL-6R (Boulanger et al., 2003). Moreover, IL-6R dimerization is primarily attributed to hydrophobic residues in the domain D2 (Varghese et al., 2002). In this study, we found that the NEGR1-IL-6R interaction was highly dependent on the D2 domain of IL-6R (Figure 5B), and the association between hyper-IL-6 and gp130 was decreased by NEGR1 overexpression (Figure 7C). Considering the highly interlocking structure of the IL-6/IL-6R/gp130 hexamer complex, we suggest that the binding of NEGR1 to the IL-6R D2 domain may hinder the formation of a functional IL-6R complex that inhibits IL-6 signaling.

There is a reciprocal link between obesity and depression that obesity increases the risk of depression and *vice versa* (Luppino et al., 2010), meanwhile, adiposity-driven inflammation contributes to depressive morbidity (Capuron et al., 2017). Multiple studies have implicated increased peripheral and central IL-6 levels in a wide spectrum of psychiatric disorders, such as major depression, schizophrenia, and autism (Quintana et al., 2013). Interestingly, while microglia directly respond to IL-6, neurons and astrocytes do not respond to IL-6 alone, highlighting the role of sIL-6R in these cells (Campbell et al., 2014). Moreover, a recent study revealed that the serum level of sIL-6R, but not IL-6 or TNF-α, is significantly associated with the pathogenesis of the treatment-resistant major depressive disorder, suggesting that IL-6 trans-signaling may be involved in the onset of this disease (Yamasaki et al., 2020).

We have previously observed that *Negr1* ^*−/−*^ mice displayed reduced neurogenesis and increased anxiety-like behavior (Noh et al., 2019). This study suggests that the affective behavior exhibited in *Negr1*-deficient mice may be linked to dysregulated levels of IL-6 and IL-6R in these mice. Given that NEGR1 is considered one of the major etiological factors of multiple mental illnesses, including major depressive disorder and autism (Deng et al., 2022), our findings may provide new insight into understanding the role of NEGR1 and IL-6 during the development of these neuropathological symptoms.

## Data Availability

The datasets presented in this study can be found in online repositories. The names of the repository/repositories and accession number(s) can be found in the article/Supplementary Material.
