# Supplementary material for: Neuronal growth regulator 1 may modulate interleukin-6 signaling in adipocytes
Source: Front Mol Biosci. 2023 Apr 28;10:1148521. doi: 10.3389/fmolb.2023.1148521 (PMC10175572; doi:10.3389/fmolb.2023.1148521)
Supplement: Supplementary file 1 [file Table1.DOCX]

Supplementary Material

Neuronal growth regulator 1 may modulate interleukin-6 signaling in adipocytes

Ara Yoo, Soojin Lee*

*** Correspondence:** Soojin Lee, leesoojin@cnu.ac.kr

##
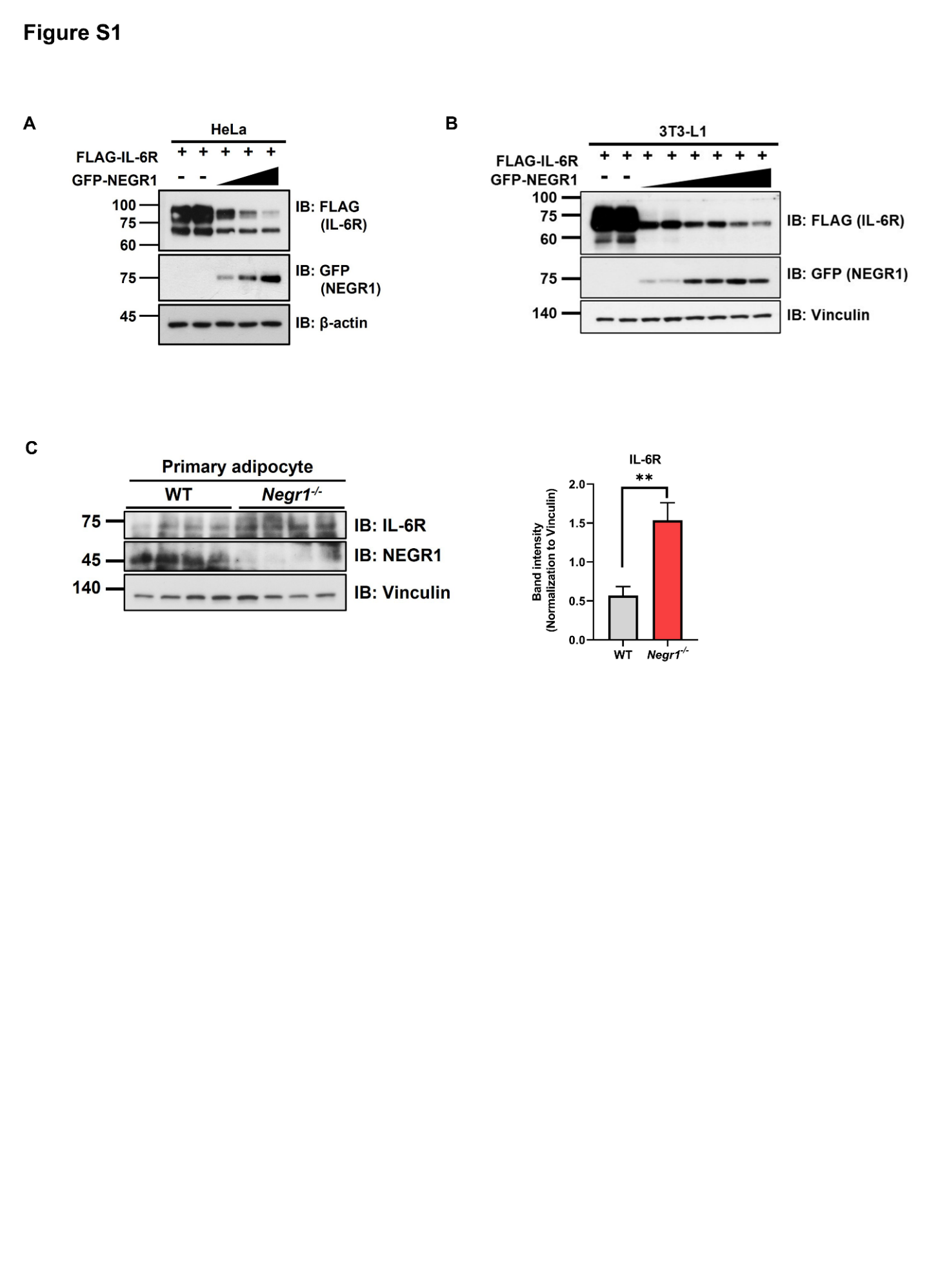
Supplementary Figures

**Supplementary Figure S1.** (A, B) IL-6R expression levels were influenced by NEGR1 co-expression. After HeLa cells (A) or 3T3-L1 cells (B) were transfected with 3FLAG-IL-6R and GFP-NEGR1 for 24 h, cells were harvested for immunoblotting. (C) Primary adipocytes were obtained from WAT of WT and *Negr1^-/-^* mice and subjected to western blotting. Band density was determined using Image J software.
